# Longitudinal Proteomic Analysis of Plasma across Healthy Pregnancies Reveals Indicators of Gestational Age

**DOI:** 10.3390/ijms23137076

**Published:** 2022-06-25

**Authors:** Elizabeth Yohannes, Danielle L. Ippolito, Jennifer R. Damicis, Elisabeth M. Dornisch, Katherine M. Leonard, Peter G. Napolitano, Nicholas Ieronimakis

**Affiliations:** 1 Department of Clinical Investigation, Madigan Army Medical Center, 9040 Jackson Ave, Tacoma, WA 98431, USA; jennifer.r.venturino.civ@mail.mil (J.R.D.); elisabeth.m.dornisch.ctr@mail.mil (E.M.D.); nicholas.m.ieronimakis.civ@mail.mil (N.I.); 2 PharmaWrite Medical Communications LLC, Princeton, NJ 08540, USA; danielle.ippolito@gmail.com; 3 Division of Maternal Fetal Medicine, Madigan Army Medical Center, 9040 Jackson Ave, Tacoma, WA 98431, USA; katherine.m.leonard9.mil@mail.mil; 4 Department of OB/GYN, University of Washington Medical Center, Seattle, WA 98195, USA; pgn@uw.edu

**Keywords:** maternal plasma proteome, LC-MS, spectral counting, ADAM12, PSG1, CSH1/2

## Abstract

Longitudinal changes in the blood proteome during gestation relate to fetal development and maternal homeostasis. Charting the maternal blood proteome in normal pregnancies is critical for establishing a baseline reference when assessing complications and disease. Using mass spectrometry-based shotgun proteomics, we surveyed the maternal plasma proteome across uncomplicated pregnancies. Results indicate a significant rise in proteins that govern placentation and are vital to the development and health of the fetus. Importantly, we uncovered proteome signatures that strongly correlated with gestational age. Fold increases and correlations between the plasma concentrations of ADAM12 (*ρ* = 0.973), PSG1 (*ρ* = 0.936), and/or CSH1/2 (*ρ* = 0.928) with gestational age were validated with ELISA. Proteomic and validation analyses demonstrate that the maternal plasma concentration of ADAM12, either independently or in combination with either PSG1 or CSH1/2, correlates with gestational age within ±8 days throughout pregnancy. These findings suggest that the gestational age in healthy pregnancies may be determined by referencing the concentration of select plasma proteins.

## 1. Introduction

Fetal development is a time of extensive growth and organogenesis. The critical measurements of growth and gestational age are typically carried out via ultrasound [1,2,3]. While ultrasound provides an accurate non-invasive means for determining fetal characteristics, it requires clinical specialization, and its accuracy declines with gestational age. Furthermore, complex patterns of biological processes that are key to normal pregnancy are orchestrated through molecular dialogs between mother, placenta, and fetus that are not completely resolved with the current form of prenatal testing [4,5,6]. Because the placenta is in direct contact with the fetus (i.e., perfused by the fetal capillary vasculature) and bathed with the maternal blood, the circulating molecular patterns in uncomplicated pregnancy reflect normal health and growth [7,8,9,10,11]. Moreover, maternal blood accessibility and its constant circulation within the placenta may provide valuable insight regarding the progression and health of the fetus in pregnancy. Although more characterization is needed for clinical translation, studies have demonstrated that the longitudinal molecular profiling of maternal blood can reflect fetal maturation, immunity, the maintenance of gestational wellbeing, and pregnancy outcomes [7,8,12,13]. 

Blood is one of the most accessed and studied biological fluid, particularly for protein analysis [14,15]. The maternal blood proteome is complex and longitudinally ill-defined, even for healthy pregnancies. Defining the normal composition of proteins in maternal blood across human gestation is critical for distinguishing signatures that predict pregnancy complications and/or detect abnormal fetal development [13]. Recently, two studies have examined longitudinal changes within the maternal blood proteome of uncomplicated pregnancies [7,8]. Both efforts utilized predefined panels targeting upward of 1310 proteins; this is a fraction of the blood proteome, estimated by some studies to contain nearly 5000 soluble proteins [16,17].

In contrast to the aforementioned studies by Romero et al. and Aghaeepour et al. [7,8], we sought to survey the composition of maternal plasma proteins across pregnancy via liquid chromatography–tandem mass spectrometry (LC-MS/MS), label-free “shotgun proteomics”. This comprehensive approach is not limited to a predefined set of targets but rather to the resolution of less abundant proteins that can be improved via depletion/selection techniques [18]. Large-scale ”precision proteomics” based on LC-MS/MS has proven to be a powerful and indispensable approach, due to its specific detection of proteins and freedom from cross-reactivity that may confound the predefined approaches [19]. Importantly, the unbiased nature of LC-MS/MS shotgun proteomics enables the identification of the variants and modifications that predefined approaches by design may not detect. For these reasons, we utilized LC-MS/MS to examine changes in the maternal blood proteome across pregnancy, using plasma samples collected from 11 participants across all three trimesters. This analysis uncovered significant changes in proteins that are important for placentation, a process vital to fetal development and health. In addition, we uncovered proteome signatures that are highly correlative with gestational age. Validation by ELISA on 12 independent participants, along with three participants examined by LC-MS/MS, substantiated that the most prominent proteins, including ADAM12, PSG1, and CSH1/2, may correspond precisely with gestational age. These findings advance our understanding of the maternal proteome and those changes that follow the natural course of pregnancy.

## 2. Results

**Figure 1:**
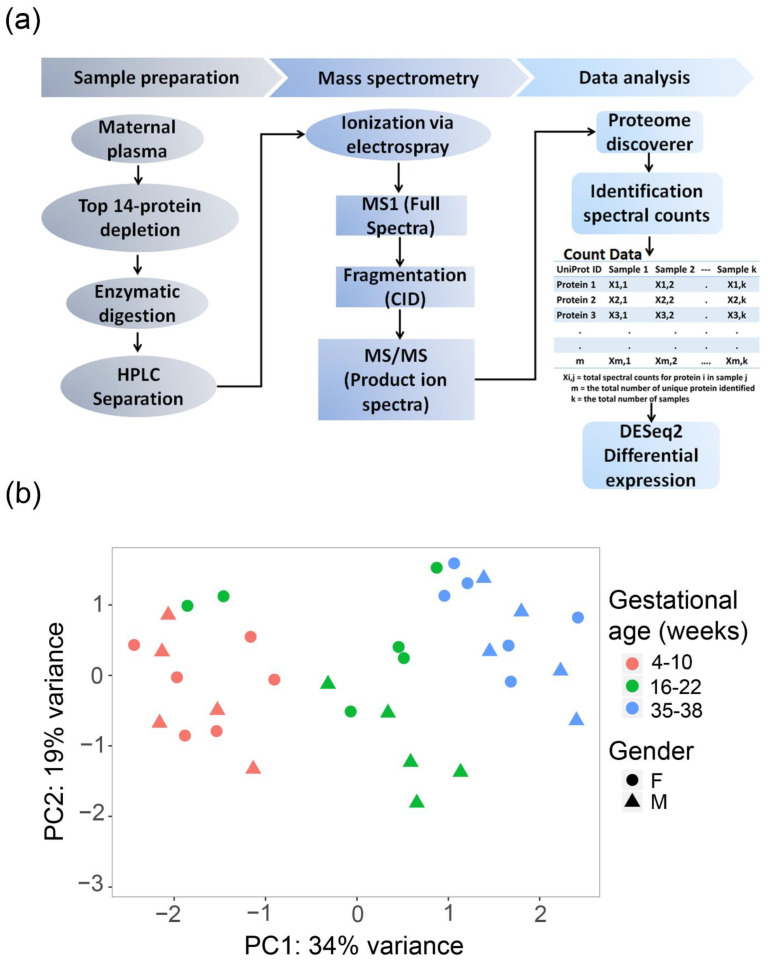
Experimental design and data summary. (**a**) Spectral-counting shotgun plasma proteomics workflow and the methods utilized to identify plasma proteins. (**b**) Principal component analysis (PCA) of longitudinal maternal plasma proteome (*n* = 11 donors) across the first (pink), second (green), and third (blue) trimesters. Data point shapes represent fetal sex (circle, female; triangle, male). Each plotted point represents an individual sample’s proteome expression profile distributed into a two-dimensional space based on the variance in proteome abundance. The axes represent the two principal components with the percentage of protein abundance variation explained by each component.

Label-free LC-MS/MS analysis, the experimental workflow design outlined in Figure 1a, revealed 5901 peptides that mapped to 282 non-redundant proteins within the three trimesters.

### 2.1. Principal Component Analysis (PCA)

Relationships among samples are highlighted by the PCA on log-transformed spectral count data for 282 proteins (Figure 1b). The primary principal component distinguishes 34% of the variance, and the second component, 19%. Samples cluster mainly according to trimester, with the exception of three second-trimester samples: two samples overlapped with the first and one with the third trimester. Overall, the PCA indicates that experimental group variations are more considerable than the within-group differences or those by fetal sex.

### 2.2. Differentially Expressed Proteins

**Figure 2:**
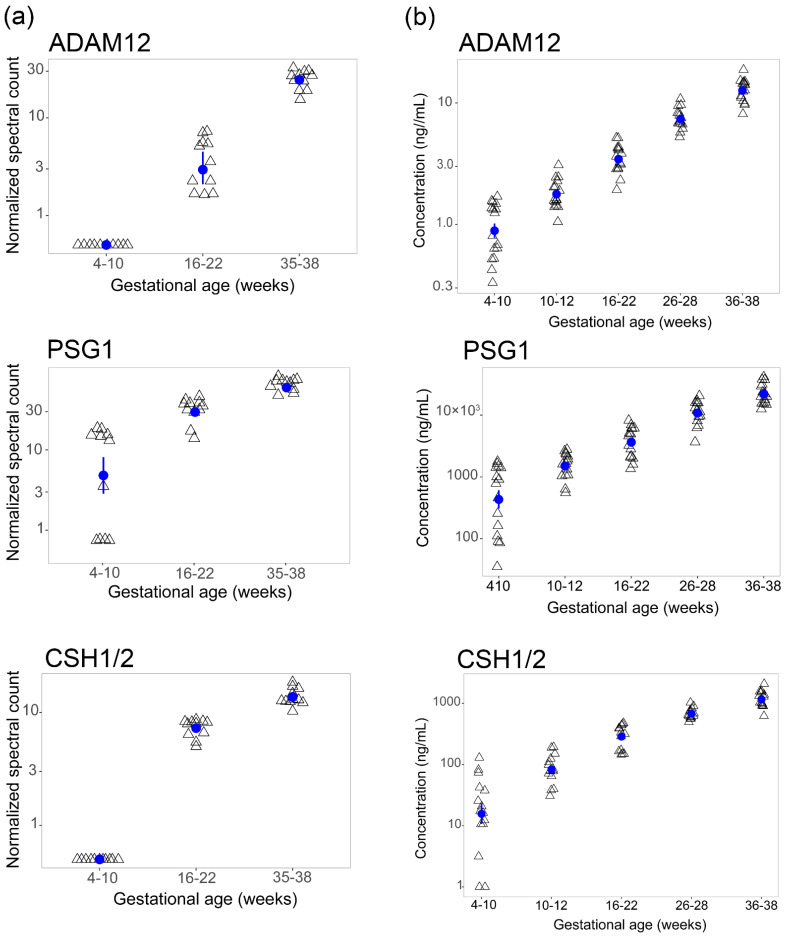
Protein abundance and validation for selected proteins: (**a**) Scatter plots of normalized spectral counts for ADAM12, PSG1, and CSH1/2 corresponding to the three gestational age timeframes (in weeks), analyzed longitudinally from each donor (*n* = 11). (**b**) Scatter plots depicting maternal plasma concentrations (ng/mL) for ADAM12, PSG1, and CSH1/2, validated by ELISA across five gestational age timeframes (in weeks) from *n* = 15 donors. Mean and standard error from the mean are represented with blue circle and bar respectively.

Once the normality of the *p*-value was confirmed, as previously described [20], *p*-values were adjusted to control for type 1 error. In accordance with the power analysis (Figure S1), proteins are considered significant if their relative abundance is LogFC ≥ 1.5 or ≤−1.5 and their adjusted *p*-value ≤ 0.05. Among the 282 proteins identified, 29 meet these criteria and are provided in Table S1. Three of these 29 proteins (ADAM12, PSG1, and CSH1/2) are significantly different between the gestational timeframes examined, consistently rising from the first to the third trimesters (Figure 2a, Table S1). Inferences for these proteins, including peptide sequences, identification scores, protein scores and coverages, and the number of protein groups and protein group accessions are provided in the database search results in Table S5.

### 2.3. Orthogonal Validation

**Figure 3:**
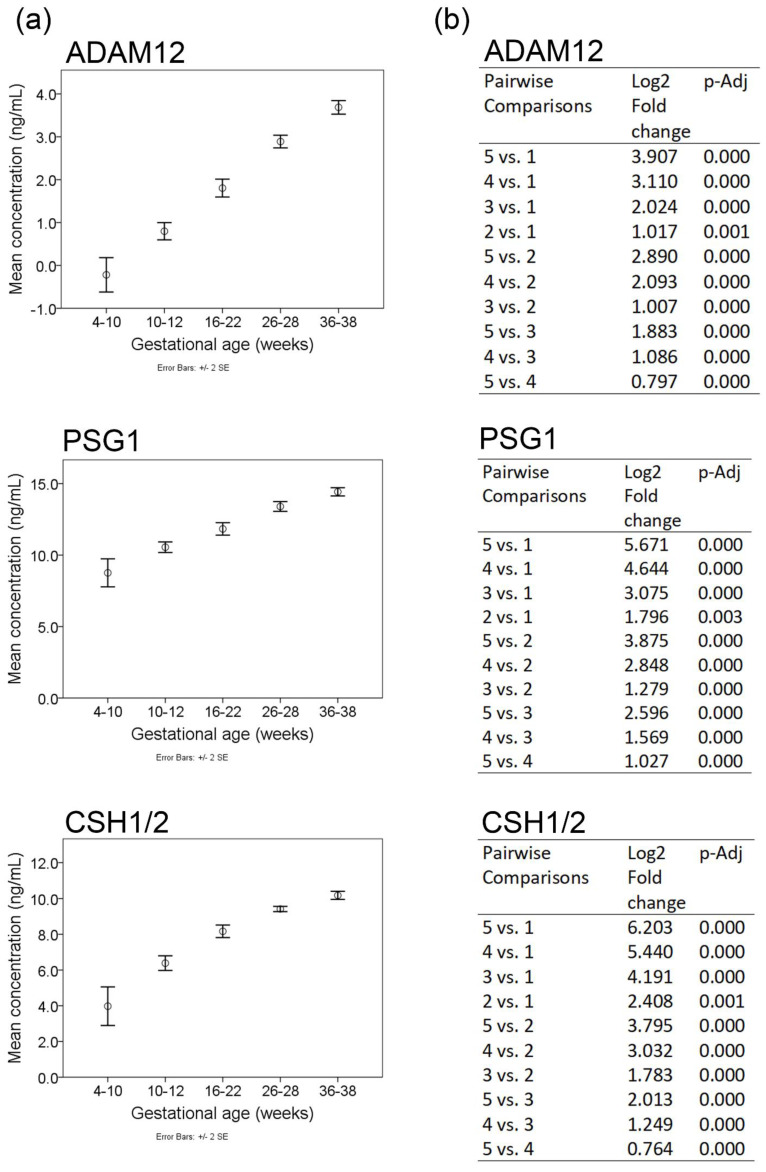
Estimates of group means and fold changes across five gestational age timeframes. (**a**) Group mean (circle) with standard errors (bars) of the mean for the log-transformed plasma concentration of ADAM12, PSG1, and CSH1/2 across the five gestational age ranges. (**b**) Table of relative fold changes and adjusted *p*-values for pairwise comparisons of the five gestational timeframes (1 = 4–10 weeks, 2 = 10–12 weeks, 3 = 16–22 weeks, 4 = 26–28 weeks, and 5 = 36–38 weeks), represented in Figure 3a.

The increasing directionality of ADAM12, PSG1, and CSH1/2 in maternal plasma was validated by ELISA in an expanded set of donor samples (*n* = 15, three of which were part of the LC-MS/MS analysis) and gestational timeframes (Figure 2b). The analysis of mean plasma concentrations of ADAM12, PSG1, and CSH1/2 was conducted by sorting the log-transformed ELISA data (Figure 2b) into five gestational age timeframes (Figure 3a). This analysis confirms significant and progressive mean differences across five gestational age timeframes for all three proteins (Figure 3b). Maximum fold changes for each target were observed at 36–38 weeks, relative to 4–10 weeks of gestation (Figure 3b).

**Figure 4:**
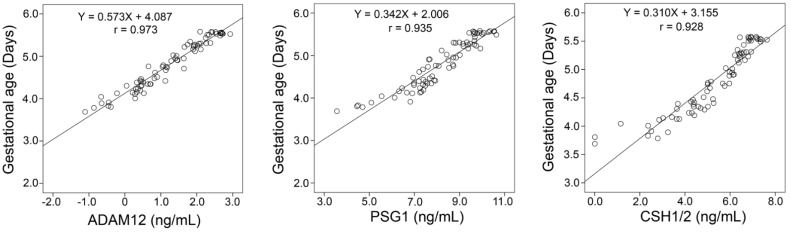
Scatter plots with regression-fit modeling. Depicted are the log-transformed maternal plasma concentrations of ADAM12, PSG1, and CSH1/2 in ng/mL (*x*-axis) from Figure 2b, plotted against each sample’s gestational age in days (*y*-axis), and also log-transformed. Pearson’s correlation coefficients are represented by the r values.

Scatterplots for log-transformed plasma concentrations of ADAM12, PSG1, and CSH1/2 and log-transformed gestational age range in days (Figure 4, S2-untransformed data) suggest a linear relationship between the plasma concentration of these proteins with the progression of pregnancy. Regression analysis supports a linear relationship between the increasing maternal plasma concentrations of ADAM12, PSG1, and/or CSH1/2 with gestational age (Figure 4). The strongest relationship is with ADAM12, followed by PSG1 and CSH1/2, as indicated by the Pearson’s correlation coefficients (*ρ* = 0.973, 0.936, and 0.928, respectively).

**Figure 5:**
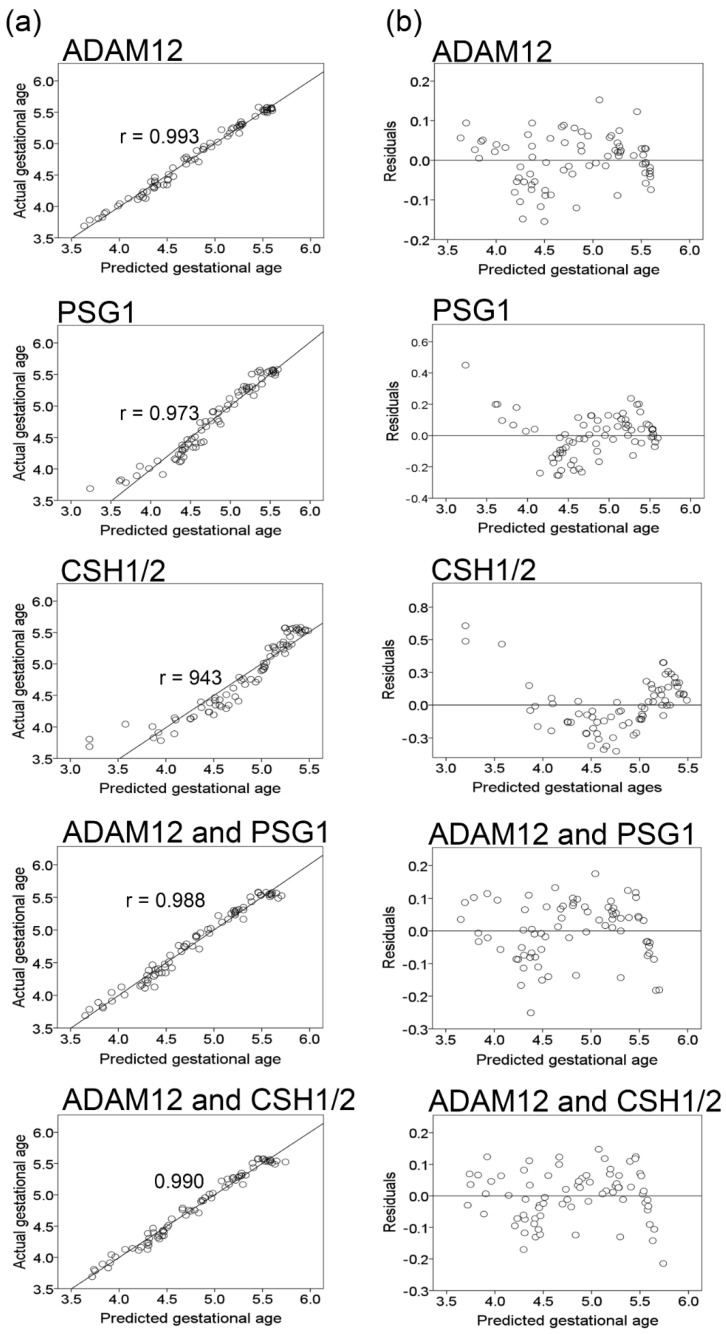
Accuracy of gestational age prediction from mixed model regression analysis. (**a**) Predicted gestational ages (*x*-axis) vs. the actual ages (*y*-axis), determined by ultrasound dating for each protein and/or combination. Values represent the days of gestation, log-transformed. (**b**) Predicted values for gestational age versus residuals for each protein. The prediction made by the model is on the *x*-axis, and the accuracy of the prediction is on the *y*-axis. The distance from the 0 line indicates how well the prediction matched the actual value.

To adjust for random effects due to repeated sampling, and to fit a model that better describes the data, linear mixed-effects modeling was utilized. Models are derived from mixed linear regression analysis, wherein the independent variables ADAM12, PSG1, and CSH1/2 are considered separately (Table S2) and/or in combination (Table S3). Estimations of the fixed effects and covariance parameters are summarized in Table S4. Based on Akaike’s information criterion (AIC), the models established using ADAM12 alone, or in combination with PSG1 or CSH1/2, outperform those derived with only PSG1, CSH1/2, or a combination of PSG1 and CSH1/2 (Tables S2 and S3). These model selections are also supported by a strong correlation between the predicted gestational ages (using ADAM12 alone, or in combination with PSG1 or CSH1/2) and gestational ages, as determined by ultrasound dating within the first 14 weeks of pregnancy (Figure 5a). Based on the residuals (Figure 5b), the best predictors of gestational age are models that include ADAM12 in combination with PSG1 or CSH1/2. The second-best predictor is the model with ADAM12 alone. The accuracy of predicting gestational age with the model derived from ADAM12, in combination with CSH1/2 or PSG1, is ±8.22 or ±8.45 days, respectively. The accuracy when using each protein alone is ±8.65 for ADAM12, ±11.12 for PSG1, and ±13.01 days for CSH1/2 alone (Tables S2 and S3).

## 3. Discussion

Understanding changes in the blood proteome across healthy pregnancies is essential for distinguishing complications. Two recent studies have reported longitudinal alterations in the maternal blood proteome for uncomplicated pregnancies [7,8]. Both studies utilized the SomaLogics SomaScan multiplexed platform, which can indirectly analyze thousands of predefined epitopes [21]. Between these two studies, 32 proteins overlapped and showed similar directionality across pregnancy [9,10]. Although the majority of proteins did not overlap, the observed differences may relate to the blood matrix used, e.g., Romero et al. analyzed serum, whereas Aghaeepour et al. examined plasma [7,8]. In terms of our results, six proteins overlapped with Romero et al. and four with Aghaeepour et al., all showing similar directionality (Table S6). These include ADAM12 and CSH1/2, but not PSG1. In contrast, our study includes validation by a secondary method (ELISA), which is recommended for confirming high-throughput results [22]; specifically, the strong correlation between the maternal plasma concentrations of ADAM12, PSG1, and/or CSH1/2 and gestational age.

Another important difference between our study and the aforementioned studies is that our samples are all from nulliparous donors. It remains unclear if parity influences the maternal proteome during gestation, yet differences have been reported in the absence of pregnancy [23]. Furthermore, it has been suggested that gravidity or parity may change the placental physiology [24]. Additional investigation is needed to determine if, during pregnancy, the maternal proteome and the relationship between maternal plasma concentrations of ADAM12, PSG1, and/or CSH1/2 and gestational age are influenced by parity.

ADAM12, PSG1, and CSH1/2 in maternal circulation have previously been studied in association with complicated pregnancies. Prior reports have correlated lower pregnant serum ADAM12 with preeclampsia, Down syndrome, and fetal growth restriction [25,26,27]. During the first trimester, longitudinal increases of ADAM12 in maternal blood are associated with placental growth and are described as essential for trophoblast fusion [28,29,30]. Therefore, the increasing trends of ADAM12 in maternal blood, observed by us and Romero et al., may reflect healthy placentation [7].

Analogous to ADAM12, the concentration of PSGs in maternal serum is lower in the case of fetal growth restriction [31,32,33,34,35] and spontaneous abortion [36,37]. A correlation between abnormally low PSG concentrations and preeclampsia was found in some studies but not in others [32,33,38,39]. Similarly, findings regarding maternal blood concentrations of CSH1/2 in relation to fetal and/or placental weight have been conflicting [40,41,42,43]. Therefore, the serum measurement of CSH1/2, in conjunction with chorionic gonadotropin in early pregnancy, has been proposed as an index of fetal and/or placenta growth and for assessing gestational age [44]. Along with those in our study, the results from Aghaeepour et al. support the predictive value of CSH1/2, in combination with additional markers, for determining gestational age at up to 30 weeks of pregnancy [8].

Although ADAM12 and CSH1/2 were described, neither Romero et al. nor Aghaeepour et al. reported longitudinal changes in PSGs (Table S6). Alongside PSG1, we observed six additional PSGs where the concentration increased with gestational age but to a lesser degree (Table S1). Over-expressed PSGs in the maternal circulation have been linked to regulating the immune and platelet responses at the maternal-fetal interface [45,46,47]. Increasing concentrations of PSGs in the maternal circulation with gestational age also reflect placental growth and health [48]. The elevation of PSG1 alongside ADAM12, and/or CSH1/2 in the maternal circulation, relative to gestational age, may be proportional to the growing placenta and essential to maintaining a healthy pregnancy to term. Deviations from such potential “baselines” may reflect complications or diseases, in consideration with pregnancy characteristics and additional biochemical testing. However, the biological significance and origin of PSGs in the maternal circulation over the course of pregnancy warrant further investigation.

This study delineates time-dependent increases in the maternal plasma concentrations of ADAM12, PSG1, and CSH1/2 over the course of normal pregnancy (Figure 2, Figure 3 and Figure 4, Tables S1–S3). Accurate dating is essential for managing delivery and, in particular, the induction of labor. Beyond 39 weeks of gestation, otherwise healthy pregnancies may develop complications and are at an increasing likelihood of cesarean delivery [49,50]. Within the initial 14 weeks of pregnancy, ultrasound dating can accurately determine gestational age within ±5–7 days [51]. Beyond this period, the accuracy of ultrasound dating gradually declines toward full-term. Despite recommendations that ultrasound dating is conducted earlier in pregnancy, cost and availability are potential barriers to access particularly in rural areas. Blood biomarkers that accurately determine gestational age may provide a cost-effective option, particularly when the availability of qualified ultrasound services is lacking.

Our results demonstrate that measuring the plasma concentrations of ADAM12, independently or in combination with PSG1 and/or CSH1/2, may determine gestational age at any trimester within ±8 days. In contrast, the accuracy of ultrasound dating beyond 22 weeks of pregnancy can be more than ±14 days [51]. For this reason, adjustments in the estimated due date are not recommended from ultrasound scans conducted beyond the first trimester [51]. Maternal plasma biomarkers may provide accurate dating in cases where pregnancy awareness occurs beyond 14 weeks of gestation or when biological processes, such as irregular menstruation and lactation, confound estimates [52]. However, these findings may not extend to pregnancy diseases that alter the maternal proteome and present later in gestation. In addition, due to the lack of diversity in our donor population (82% white) and our small sampling, these findings may not translate to all pregnancies or account for differences related to fetal sex-. Larger studies are needed to determine and advance the utility of these or other maternal plasma proteins for estimating gestational age within a broader population and among multiparous women.

## 4. Materials and Methods

### 4.1. Patient Selection and Clinical Samples

Study approval was granted by the Institutional Review Board. Maternal blood was collected longitudinally from pregnant donors using EDTA purple-top tubes and plasma was processed as previously described [53]. Briefly, blood was centrifuged at 1500× *g* for 15 min at 4 °C to separate the plasma, which received protease inhibitor (Roche Life Science, Indianapolis, IN, USA) prior to aliquoting and storage at −150 °C. All samples used in this study were from nulliparous and uncomplicated pregnancies and were taken from donors who self-reported as being free of tobacco, alcohol, and drug use. The pregnancy characteristics are summarized in Table 1.

### 4.2. Experimental Design and Statistical Rationale

To estimate the number of samples, a power analysis was performed using a pilot shotgun proteomics data set from each trimester, an open-source DEseq2, and the RnaSeqSampleSize package (https://www.bioconductor.org) (accessed on 22 November 2017). The power curves per hypothesis of the negative binomial distribution test were generated as a function of the sample size required to detect log2 fold changes of 1.5, 2, and 3, at the adjusted *p*-values of 0.05, 0.01, and 0.001 (Figure S1).

### 4.3. Label-Free Shotgun LC-MS/MS

Plasma specimens from 11 participants across the first, second, and third trimesters were analyzed using the general shotgun proteomics workflow shown in Figure 1a. Briefly, plasma samples were depleted of 14 highly abundant proteins using affinity removal spin cartridges (Hu-14, Agilent Technologies, Santa Clara, CA, USA). The samples were concentrated and buffer-exchanged with 6 M urea and 25 mM Trizma pH 8.0, to a final volume of 50 μL, using a 3 kDa molecular weight cutoff filter (Millipore Sigma, Burlington, MA, USA). Samples were normalized via BCA assay (Thermo Fisher Scientific, Waltham, MA, USA); subsequently, 10 µg of total protein from each sample was reduced and alkylated with DTT (15 mM) and iodoacetamide (40 mM), respectively. Proteolytic digestion was followed by LC-MS/MS analysis, performed using the general workflow previously described in detail [20].

### 4.4. Protein Identification and Data Search Parameters

Raw data files were imported into Proteome Discoverer, version 1.4 (Thermo Fisher Scientific, Waltham, MA, USA), and the peak lists were extracted. The resulting peak-list files were searched against the Swiss-Prot.fasta human database (covering 20,183 sequences), which was downloaded on 23 March 2017 from UniProt (http://www.uniprot.org/) (accessed on 21 November 2017) to the local server, using the general workflow previously described in detail [20]. The peptide-spectrum match (PSM) is considered correct if it achieved an estimated q-value (minimal false discovery rate) of 0.01 or less. For protein identification, a minimum of two peptides with delta Cn (delta correlation) ≤0.05 and with high confidence based on q ≤ 0.01 were utilized to ensure the protein level stringency. 

### 4.5. Quantitation and Statistical Analysis

The relative abundance for identified proteins was measured on the basis of the spectral count, defined by the total number of identified peptide spectra matched to the protein of interest, including those that were redundantly identified. Differential expression analyses across the three gestation periods were performed using DESeq2, a package for the R statistical environment [54] (http://www.biocondactor.org) (accessed on 22 November 2017). The method and the workflow used to test for differential expression were conducted as previously described [20]. Data extracted for the proteome results included log2 fold changes, *p*-values, and adjusted *p*-values, while differentially expressed proteins were filtered with a Benjamini Hochberg adjusted *p*-value ≤ 0.05 and are summarized in Table S1.

### 4.6. High-Dimensional Data Visualization

The count data was first log-transformed, then principal components of the samples were visualized by performing PCA (using the plotPCA Bioconductor package) analysis.

### 4.7. Enzyme-Linked Immunosorbent Assay (ELISA)

Plasma specimens collected from pregnant women (*n* = 15) longitudinally across five gestational age ranges (4–10, 10–12, 16–22, 26–28, and 36–38 weeks) were analyzed by ELISA, according to the manufacturer’s protocol (R&D Systems, Inc., Minneapolis MN, and DRG International, Inc., Springfield, NJ, USA). Three donor samples that were also examined by LC-MS/MS were included in this analysis, due to their availability of plasma for all five timeframes. 

### 4.8. ELISA Data Analysis

The Statistical Package for the Social Sciences (SPSS), version 24.0.0.0 (https://www.ibm.com) (accessed on 22 February 2019), and lem4 (http://www.biocondactor.org) (accessed on 30 April 2020) were used for data analysis. Mean differences across the five gestational time points were assessed using linear mixed-effects models with a correlation structure. Evidence of the relationship between estimated gestational age and the maternal plasma concentration of ADAM12, PSG1, and CSH1/2 was examined by linear mixed-model regression analysis. To account for within-subject clustering or correlations induced by repeated measurement, the analysis was modeled with random intercept or with slope alone, as well as both random intercept and slope. Prediction models for a standalone target were compared with models for combinations of targets by computing the Akaike information criterion (AIC) for each model in a forward model selection scheme, until the addition of extra variables no longer led to an improvement, i.e., a reduction in the AIC.

## Figures and Tables

**Table 1:** Pregnancy characteristics of study samples. Longitudinal plasma samples from 11 donors, used for shotgun proteomics discovery via LC-MS/MS. Plasma samples from 12 independent participants, along with 3 examined with LC-MS/MS, were analyzed by ELISA.

Characteristics of the Study Population	Mean (IQR) or % (n) *
Maternal Age (years)	26.13 ± 1 (22–29)
Body mass index (BMI), kg/m^2^	25.21 ± 0.89 (24–30)
Parity	0
Race	
White (Caucasian)	82.6% (19)
African American	8.7% (2)
Asian	4.3% (1)
Other	4.3% (1)
Type of delivery	
Vaginal	69.6% (16)
Cesarean	30% (7)
Birthweight, Kg	3.57 ± 0.14 (3.26–3.76)
Gestational age (weeks)	39.72 ± 0.24 (39.14–40.75)

* Data are presented as a percentage (number) for categorical variables and mean (IQR) for continuous variables. IQR: Interquartile range.

## Data Availability

The raw mass spectrometry data sets are deposited at URL (ftp://massive.ucsd.edu//MSV000089320) (accessed on 27 April 2022) on the ProteomeXchange consortium.

## Supplementary Materials

The following supporting information can be downloaded at: https://www.mdpi.com/article/10.3390/ijms23137076/s1.
