# Supplementary material for: Longitudinal Proteomic Analysis of Plasma across Healthy Pregnancies Reveals Indicators of Gestational Age
Source: Int J Mol Sci. 2022 Jun 25;23(13):7076. doi: 10.3390/ijms23137076 (PMC9266720; doi:10.3390/ijms23137076)
Supplement: Supplementary file 1 [file ijms-23-07076-s001.zip › ijms-1724493-supplementary-english.pdf]

Longitudinal proteomic analysis of plasma across healthy pregnancies reveals indicators of gestational age

Elizabeth Yohannes [1,*], Danielle L. Ippolito [2], Jennifer R. Damicis [1], Elisabeth M. Dornisch [1], Katherine M. Leonard [3], Peter G. Napolitano [4] and Nicholas Ieronimakis [1,3]

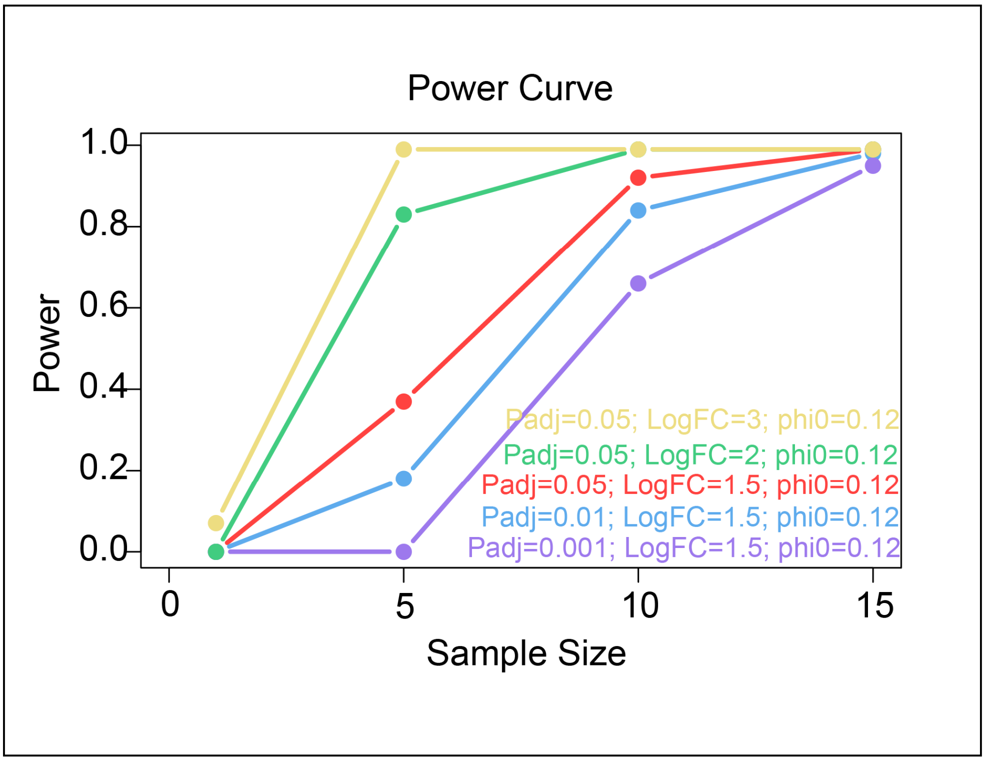

Figure S1: The relationship between power and sample size, at Padj = 0.001, 0.01 or 0.05 for logFC = 1.5, 2, or 3, in protein expression. It is clear from these curves that with the data median dispersion (dispersion = 0.12 from our pilot study), significance level, and sample size have a profound effect on power. As clinical sample numbers were limited, the power curve at *p*-adjusted = 0.05 was an appropriate choice for our study. Using this curve, it was estimated that biological replicates of ten, per experimental group, result in >= 80% of chance of detecting a fold change of 1.5- and above. Thus, we designed this study, taking *n* = 11 for each experimental group.

Table S1: Differential maternal plasma proteome in the normal pregnancy

Accession	Protein identity	Log2 Fold change			p-adj			Bio-process
		2nd/1st	3rd/1st	3rd/2nd	2nd/1st	3rd/1st a	3rd/2nd	
O43184	Disintegrin and metalloproteinase domain-containing protein 12 OS=Homo sapiens GN=ADAM12 PE=1 SV=3 - [ADAM12_HUMAN]	3.91	7.06	3.15	4.47E-06	3.52E-22	1.43E-12	Proteolysis, cell adhesion, integrin-mediated signaling pathway, myoblast fusion.
P0DML2	Chorionic somatomammotropin hormone 1 OS=Homo sapiens GN=CSH1 PE=1 SV=1 - [CSH1_HUMAN]	5.22	6.17	0.95	3.38E-13	7.50E-20	6.24E-03	JAK-STAT cascade, involved in the growth-hormone signaling pathway.
Q9UIQ6	Leucyl-cysteinyl aminopeptidase OS=Homo sapiens GN=LNPEP PE=1 SV=3 - [LCAP_HUMAN]		6.85	6.85		5.09E-15	2.85E-14	Female pregnancy, antigen processing, and the presentation of exogenous peptide antigen via MHC class I, regulation of blood pressure, SMAD protein signal transduction.

Q13219	Pappalysin-1 OS=Homo sapiens GN=PAPPA PE=1 SV=3 - [PAPP1_HUMAN]	5.31	6.59		9.90E-09	3.56 E-14		Female pregnancy, response to follicle-stimulating hormone.
P11464	Pregnancy-specific beta-1-glycoprotein 1 OS=Homo sapiens GN=PSG1 PE=1 SV=1 - [PSG1_HUMAN]	1.8	2.86		3.2E-04	2.82E-11		Female pregnancy, defense response.
P11465	Pregnancy-specific beta-1-glycoprotein 2 OS=Homo sapiens GN=PSG2 PE=2 SV=2 - [PSG2_HUMAN]	2.18	2.60		2.7E-04	2.05E-10		Female pregnancy, cell migration.
Q9UQ72	Pregnancy-specific beta-1-glycoprotein 11 OS=Homo sapiens GN=PSG11 PE=2 SV=3 - [PSG11_HUMAN]		6.52	4.28		5.06E-09	7.3E-04	Female pregnancy.
Q04756	Hepatocyte growth factor activator OS=Homo sapiens GN=HGFAC PE=1 SV=1 - [HGFA_HUMAN]		-1.37	-0.95		1.39E-07	4.1E-03	Proteolysis.
P13727	Bone marrow proteoglycan OS=Homo sapiens GN=PRG2 PE=1 SV=2 - [PRG2_HUMAN]	2.09	2.15		2.2E-06	2.80E-07		Immune response, regulation of cytokine biosynthetic process.

P07333	Macrophage colony-stimulating factor 1 receptor OS=Homo sapiens GN=CSF1R PE=1 SV=2 - [CSF1R_HUMAN]	3.05	5.33		0.045	8.59E-07		Inflammatory response, positive regulation of protein phosphorylation, signal transduction, transmembrane receptor protein tyrosine kinase signaling pathway.
Q00888	Pregnancy-specific beta-1-glycoprotein 4 OS=Homo sapiens GN=PSG4 PE=2 SV=3 - [PSG4_HUMAN]		6.96	4.11		8.59E-07	0.016	Female pregnancy.
P09466	Glycodelin OS=Homo sapiens GN=PAEP PE=1 SV=2 - [PAEP_HUMAN]	-5.06	-5.09		4.47E-06	1.15E-06		Transport, apoptotic process, multicellular organism development.
Q16557	Pregnancy-specific beta-1-glycoprotein 3 OS=Homo sapiens GN=PSG3 PE=2 SV=2 - [PSG3_HUMAN]	6.54	4.78			2.31E-06	4.10E-03	Female pregnancy, defense response.
Q00887	Pregnancy-specific beta-1-glycoprotein 9 OS=Homo sapiens	1.22	1.85		0.013	4.26E-06		Female pregnancy.

	GN=PSG9 PE=2 SV=2 - [PSG9_HUMAN]							
Q13822	Ectonucleotide pyrophosphatase/phosphodiesterase family member 2 OS=Homo sapiens GN=ENPP2 PE=1 SV=3 - [ENPP2_HUMAN]		2.58	1.7		6.07E-05	7.3E-03	Phosphate-containing compound metabolic process, receptor-mediated endocytosis, chemotaxis, immune response.
P03951	Coagulation factor XI OS=Homo sapiens GN=F11 PE=1 SV=1 - [FA11_HUMAN]		-1.55			2.2E-04		Proteolysis, blood coagulation, intrinsic pathway, plasminogen activation, positive regulation of fibrinolysis.
Q00889	Pregnancy-specific beta-1-glycoprotein 6 OS=Homo sapiens GN=PSG6 PE=2 SV=1 - [PSG6_HUMAN]	3.52	6.12	2.61	8.0E-03	6.39E-04	4.3E-02	Female pregnancy.
P04275	von Willebrand factor OS=Homo sapiens GN=VWF PE=1 SV=4 - [VWF_HUMAN]		2.46	1.96		7.4E-04	3.7E-02	Platelet degranulation, cell adhesion, hemostasis, response to wounding, platelet activation,

								extracellular matrix organization.
Q6EMK4	Vasorin OS=Homo sapiens GN=VASN PE=1 SV=1 - [VASN_HUMAN]		-3.68			8.70E-04		Negative regulation of epithelial to mesenchymal transition, the negative regulation of transforming the growth factor beta receptor signaling pathway, cellular response to redox state.
Q9Y5Y7	Lymphatic vessel endothelial hyaluronic acid receptor 1 OS=Homo sapiens GN=LYVE1 PE=1 SV=2 - [LYVE1_HUMAN]		-3.68			1.61E-03		Glycosaminoglycan catabolic process, signal transduction, response to wounding, anatomical structure morphogenesis.
P01009	Alpha-1-antitrypsin OS=Homo sapiens GN=SERPINA1 PE=1 SV=3 - [A1AT_HUMAN]		2.0			2.7E-03		Platelet degranulation, ER to Golgi vesicle-mediated transport, acute-phase response.

Q15485	Ficolin-2 OS=Homo sapiens GN=FCN2 PE=1 SV=2 - [FCN2_HUMAN]	-2.29	-2.65			3.8E-03	4.6E-02	Complement activation of a defense response bacterium.
P0DN86	Choriogonadotropin subunit beta 3 OS=Homo sapiens GN=CGB3 PE=1 SV=1 - [CGB3_HUMAN]		-1.95			5.12E-03		
P54108	Cysteine-rich secretory protein 3 OS=Homo sapiens GN=CRISP3 PE=1 SV=1 - [CRIS3_HUMAN]		-1.41			5.29E-03		Defense response, innate immune response.
P13796	Plastin-2 OS=Homo sapiens GN=LCP1 PE=1 SV=6 - [PLSL_HUMAN]		-2.74			6.16E-03		T-cell activation involved in the immune response, protein kinase A signaling, and cell migration.
Q15848	Adiponectin OS=Homo sapiens GN=ADIPOQ PE=1 SV=1 - [ADIPO_HUMAN]		-3.79			7.36E-03		Response to hypoxia, positive regulation of protein phosphorylation, and glucose metabolic process.
O75015	Low-affinity immunoglobulin gamma Fc region receptor III-B		-3.64			9.40E-03		

	OS=Homo sapiens GN=FCGR3B PE=1 SV=2 - [FCG3B_HUMAN]							
Q4LDE5	Sushi, von Willebrand factor type A, EGF and pentraxin domain-containing protein 1 OS=Homo sapiens GN=SVEP1 PE=1 SV=3 - [SVEP1_HUMAN]		4.89	4.90		1.0E-02	4.7E-02	Cell adhesion.
Q9BXR6	Complement factor H-related protein 5 OS=Homo sapiens GN=CFHR5 PE=1 SV=1 - [FHR5_HUMAN]		-1.79			2.6E-02		Complement activation, alternative pathway.

[a] Ranked based on the lowest to highest *p*-adj for the third vs the first trimester.

Table S2: Linear mixed-model regression analysis model summary, describing the relationship between gestational age and the maternal plasma levels of ADAM12, PSG1, or CSH1/2 over the course of a normal pregnancy.

Parameter	Slope		Intercept		p-value	AIC	SE_{Pred}
	Estimate	SE	Estimate	SE			
ADAM12	0.574	0.019	4.087	0.039	0.000	-107.50	8.65
PSG1	0.371	0.011	1.769	0.101	0.000	-41.54	11.13
CSH1/2	0.318	0.014	3.120	0.078	0.000	-13.54	13.02

SE, standard error; AIC, Akaike's information criterion; SEpred, standard error of the prediction estimate.

Table S3: Accuracy of prediction models established with different combinations of targets

Targets	Regression Model	AIC	SE_{pred}
ADAM12, PSG1	GA = 3.59 + 0.244ADAM12 +0.081PSG1 + 0.02ADAM12 * PSG1	-107.63	8.46
ADAM12, CSH1/2	GA = 3.79 + 0.176ADAM12 + 0.095CSH1/2 + 0.035ADAM12 * CSH1/2	-118.73	8.22
PSG1, CSH1/2	GA = 3.63 – 0.094CSH1/2 + 0.036PSG1 * CSH1/2	-107.37	8.60

AIC, Akaike's Information Criterion; SEpred, standard error of the prediction estimate

Table S4: Parameters of the mixed model analysis results.

ADAM12 alone

Estimates of Fixed Effects[a]

Parameter	Estimate	Std. Error	df	t	Sig.	95% Confidence Interval Lower Bound	95% Confidence Interval Upper Bound
Intercept	4.111013	.029408	24.844	139.791	.000	4.050427	4.171600
ADAM12	.557664	.011549	60.789	48.287	.000	.534569	.580759

Estimates of Covariance Parameters[a]

Parameter	Estimate	Std. Error	Wald Z	Sig.	95% Confidence Interval Lower Bound	95% Confidence Interval Upper Bound
Residual	.010112	.001849	5.469	.000	.007067	.014471
Intercept Variance [subject = ID]	.007865	.003653	2.153	.031	.003165	.019547

PSG alone

Estimates of Fixed Effects[a]

Parameter	Estimate	Std. Error	df	t	Sig.	95% Confidence Interval Lower Bound	95% Confidence Interval Upper Bound
Intercept	1.769820	.101171	74.898	17.493	.000	1.568272	1.971368
PSG1	.371048	.011291	62.026	32.862	.000	.348478	.393618

Estimates of Covariance Parameters[a]

Parameter	Estimate	Std. Error	Wald Z	Sig.	95% Confidence Interval Lower Bound	95% Confidence Interval Upper Bound
Residual	.021064	.003867	5.447	.000	.014699	.030186
Intercept Variance [subject = ID]	.021490	.009637	2.230	.026	.008923	.051757

CSH1/2 alone

Estimates of Fixed Effects[a]

Parameter	Estimate	Std. Error	df	t	Sig.	95% Confidence Interval Lower Bound	95% Confidence Interval Upper Bound
Intercept	3.120282	.078002	74.930	40.002	.000	2.964891	3.275673
CSH1	.318547	.013573	63.818	23.470	.000	.291432	.345663

Estimates of Covariance Parameters[a]

Parameter	Estimate	Std. Error	Wald Z	Sig.	95% Confidence Interval Lower Bound	95% Confidence Interval Upper Bound
Residual	.039022	.007190	5.427	.000	.027194	.055996
Intercept Variance [subject = ID]	.006310	.005581	1.131	.258	.001115	.035720

ADAM12 and PSG1 in combination

Estimates of Fixed Effects[a]

Parameter	Estimate	Std. Error	df	t	Sig.	95% Confidence Interval Lower Bound	95% Confidence Interval Upper Bound
Intercept	3.592862	.197365	47.075	18.204	.000	3.195833	3.989892
ADAM12	.244189	.087384	69.853	2.794	.007	.069901	.418477
PSG1	.081490	.030645	47.344	2.659	.011	.019853	.143127
ADAM12 * PSG1	.020706	.006986	68.182	2.964	.004	.006765	.034646

Estimates of Covariance Parameters[a]

Parameter	Estimate	Std. Error	Wald Z	Sig.	95% Confidence Interval Lower Bound	95% Confidence Interval Upper Bound
Residual	.009072	.001663	5.456	.000	.006334	.012993
Intercept Variance [subject = ID]	.005172	.002616	1.977	.048	.001919	.013938

ADAM12 and CSH1/2 in combination

Estimates of Fixed Effects[a]

Parameter	Estimate	Std. Error	df	t	Sig.	95% Confidence Interval Lower Bound	95% Confidence Interval Upper Bound
Intercept	3.791326	.076488	74.966	49.568	.000	3.638953	3.943699
ADAM12	.176045	.074860	72.621	2.352	.021	.026835	.325255
CSH1	.095450	.021517	73.143	4.436	.000	.052568	.138332
ADAM12 * CSH1	.035328	.007799	68.908	4.530	.000	.019769	.050888

Estimates of Covariance Parameters[a]

Parameter	Estimate	Std. Error	Wald Z	Sig.	95% Confidence Interval Lower Bound	95% Confidence Interval Upper Bound
Residual	.007321	.001339	5.469	.000	.005116	.010477
Intercept Variance [subject = .ID]	.006389	.002899	2.204	.028	.002625	.015547

CSH1/2 and PSG1 in combination

Estimates of Fixed Effects[a]

Parameter	Estimate	Std. Error	df	t	Sig.	95% Confidence Interval Lower Bound	95% Confidence Interval Upper Bound
Intercept	3.628051	.056454	74.458	64.266	.000	3.515576	3.740526
CSH1	-.093668	.029330	65.990	-3.194	.002	-.152228	-.035108
PSG1 * CSH1	.036565	.002497	65.684	14.645	.000	.031580	.041551

Estimates of Covariance Parameters[a]

Parameter	Estimate	Std. Error	Wald Z	Sig.	95% Confidence Interval Lower Bound	95% Confidence Interval Upper Bound
Residual	.008731	.001598	5.464	.000	.006099	.012498
Intercept Variance [subject = ID]	.007720	.003507	2.201	.028	.003169	.018808

a. Dependent Variable: Gestational age.
